# Retraction: TRPM8 facilitates proliferation and immune evasion of esophageal cancer cells

**DOI:** 10.1042/BSR20191878_RET

**Published:** 2026-09-09

**Authors:** Xinyan Lan, Jun Zhao, Chunjing Song, Qiuxiang Yuan, Xiaojun Liu

**Affiliations:** 1 Department of Thoracic Surgery, Qingdao Chengyang District People's Hospital, NO. 600 Changcheng Road, Chengyang District, Qingdao 266109, China; 2 Department of Urinary Surgery, Qingdao Chengyang District People's Hospital, NO. 600 Changcheng Road, Chengyang District, Qingdao 266109, China; 3 Department of Gastroenterology, Qingdao Chengyang District People's Hospital, NO. 600 Changcheng Road, Chengyang District, Qingdao 266109, China

**Keywords:** Esophageal cancer, Immune evasion, Proliferation, TRPM8

This article is being retracted from *Bioscience Reports* at the request of the Editor-in-Chief and the Editorial Board. This follows an internal investigation that identified multiple similarities within the article and with another article published by different authors. Specifically, these include high similarity between:

- The Figure 1J GAPDH/empty vector band and the Figure 3F GAPDH/TRPM8 OE band
- The Figure 1J GAPDH/TRPM8 OE band and the Figure 3E GAPDH/control siRNA band
- The first panel of Figure 2E and the Figure 2C Inhibitor NC panel of Kuai et al. 2022 (DOI: https://doi.org/10.2147/CCID.S367347)
- The third panel of Figure 2E and the Figure 2D miR-23b-3p inhibitor panel of Kuai et al. 2022

The authors have been contacted with regard to the retraction and have not responded to the Journal's queries or to the concerns. Given the extent of the issues raised, the Editorial Board stands by the decision to retract the article.

